# Plasma lipidomic analysis reveals strong similarities between lipid fingerprints in human, hamster and mouse compared to other animal species

**DOI:** 10.1038/s41598-018-34329-3

**Published:** 2018-10-26

**Authors:** Zied Kaabia, Julie Poirier, Michelle Moughaizel, Audrey Aguesse, Stéphanie Billon-Crossouard, Fanta Fall, Manon Durand, Elie Dagher, Michel Krempf, Mikaël Croyal

**Affiliations:** 1 Mass Spectrometry Core facility of West Human Nutrition Research Center (CRNHO), Hotel Dieu Hospital, Nantes, France; 2 Inra, UMR 1280, Physiologie des Adaptations Nutritionnelles, Nantes, France; 3 Department of Pathology, Oniris, Nantes Atlantic College of Veterinary Medicine Food Science and Engineering, Nantes, France; 4 Department of Endocrinology, Metabolic diseases and Nutrition, G and R Laennec Hospital, Nantes, France

## Abstract

Cardiovascular diseases are often associated with impaired lipid metabolism. Animal models are useful for deciphering the physiological mechanisms underlying these pathologies. However, lipid metabolism is contrasted between species limiting the transposition of findings from animals to human. Hence, we aimed to compare extended lipid profiles of several animal species to bring new insights in animal model selections. Human lipid phenotype was compared with those of 10 animal species. Standard plasma lipids and lipoprotein profiles were obtained by usual methods and lipidomic analysis was conducted by liquid chromatography-high-resolution mass spectrometry (LC-HRMS). As anticipated, we found contrasted lipid profiles between species. Some of them exhibited similar plasma lipids to human (non-human primate, rat, hamster, pig), but only usual lipid profiles of pigs were superimposable with human. LC-HRMS analyses allowed the identification of 106 other molecular species of lipids, common to all samples and belonging to major lipid families. Multivariate analyses clearly showed that hamster and, in a lower extent mouse, exhibited close lipid fingerprints to that of human. Besides, several lipid candidates that were previously reported to study cardiovascular diseases ranged similarly in human and hamster. Hence, hamster appeared to be the best option to study physiological disturbances related to cardiovascular diseases.

## Introduction

Atherothrombotic cardiovascular diseases (ACVD) are the leading cause of death worldwide and are widely associated with lipid disturbances^1–3^. Animal models are essential to understand the physiological mechanisms of ACVD in humans. However, lipoprotein metabolism and vascular physiology are often contrasted between species limiting the transposition of findings from animals to human^4,5^. Such differences should be considered for animal model selection and for data understanding within a realistic extrapolation framework for a better assessment of disturbances involved in humans^6^.

Most of lipid comparisons between species are primarily focused on plasma lipoprotein profiles and major lipid components such as total cholesterol (TC) and triglycerides (TG)^7^. However, other circulating lipids within or out lipoproteins could also potentially be involved in the process of atherosclerotic lesions. In that respect, glycerophospholipids and sphingolipids have been shown to be significantly altered during the atherosclerotic progression in apoE^−/−^ mice^8^, and other studies showed that elevated concentrations of lysophosphatidylcholines (LPC) and sphingomyelins (SM) were also correlated with atherosclerosis development^9,10^. These findings have been recently supported by human prospective ACVD outcome studies^11^.

Here we explored an extended lipid profile of nine animal species including non-human primate, dog, cat, pig, horse, bovine, hamster, mouse and rat along with human. Some of these species were already described as potential models for human ACVD^12^. We aimed to investigate both global (non-targeted) and specific (targeted) lipid profiles in those species to create global lipid fingerprints and then assess the similarity and differences compared with humans.

## Results

### Lipoprotein profiles

**Figure 1:**
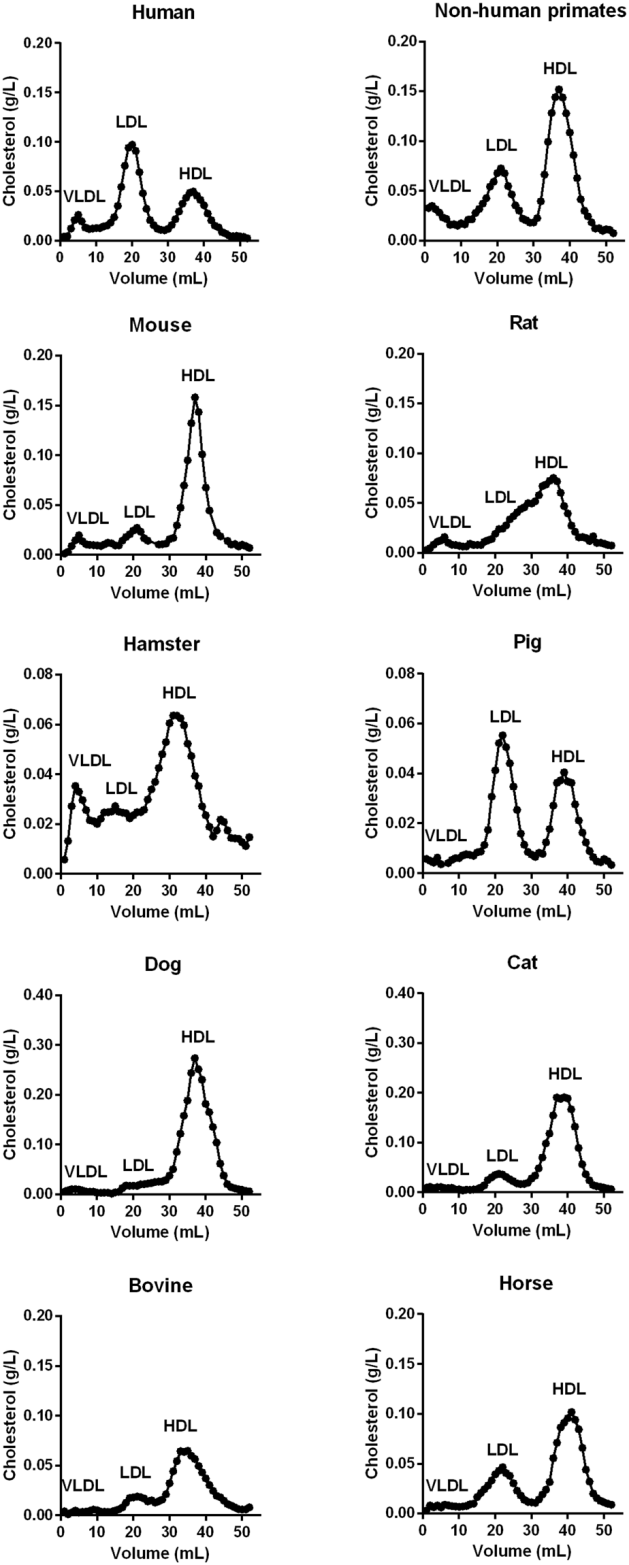
Lipoprotein profiles of studied species. The mean lipoprotein profiles (n = 6) were obtained by fast performance liquid chromatography (FPLC).

Plasma lipids were measured for the different animal species and are displayed in Table 1. In contrast to human, FPLC profiles showed most of species carried cholesterol primarily within HDL particles (Fig. 1). For NHP, mouse, dog, cat, horse and cow, LDL cholesterol was markedly lower than HDL cholesterol (Fig. 1). In contrast, pig displayed a lipoprotein profile comparable to human characterized by elevated LDL cholesterol. Of note, hamster and rat exhibited similar lipoprotein profiles characterized by a strong overlap between both LDL and HDL particles (Fig. 1). Finally, triglyceride-rich lipoproteins (VLDL) were mainly detected in human and non-herbivorous species.

**Table 1:** Plasma lipid concentrations in human and animal plasma.

Species	Strain	n	TC	TG	CE	PL
Human	*Caucasian*	6	201 ± 34	62 ± 13	164 ± 28	231 ± 19
NHP	*Cynomolgus*	6	126 ± 8	56 ± 10	106 ± 7	212 ± 33
Mouse	*C57BL/6*	6	135 ± 7	111 ± 27	114 ± 6	261 ± 17
Rat	*Wistar*	6	133 ± 8	64 ± 13	111 ± 8	223 ± 15
Hamster	*Golden Syrian*	6	172 ± 33	87 ± 25	134 ± 28	263 ± 30
Pig	*West white pork*	6	133 ± 15	43 ± 8	116 ± 14	129 ± 13
Bovine	*Holstein*	6	99 ± 34	11 ± 4	84 ± 31	89 ± 22
Horse	*Lusitanian*	6	136 ± 18	29 ± 16	114 ± 16	186 ± 26
Dog	*Beagle*	6	320 ± 61	39 ± 6	266 ± 45	344 ± 35
Cat	*European*	6	248 ± 43	31 ± 9	209 ± 38	231 ± 18

Values are mean ± standard deviation (3 males, 3 females) and are expressed in mg/dL.

TC, total cholesterol; TG, triglycerides; CL, cholesterol esters; PL, phospholipids.

### Non-targeted lipidomics

**Figure 2:**
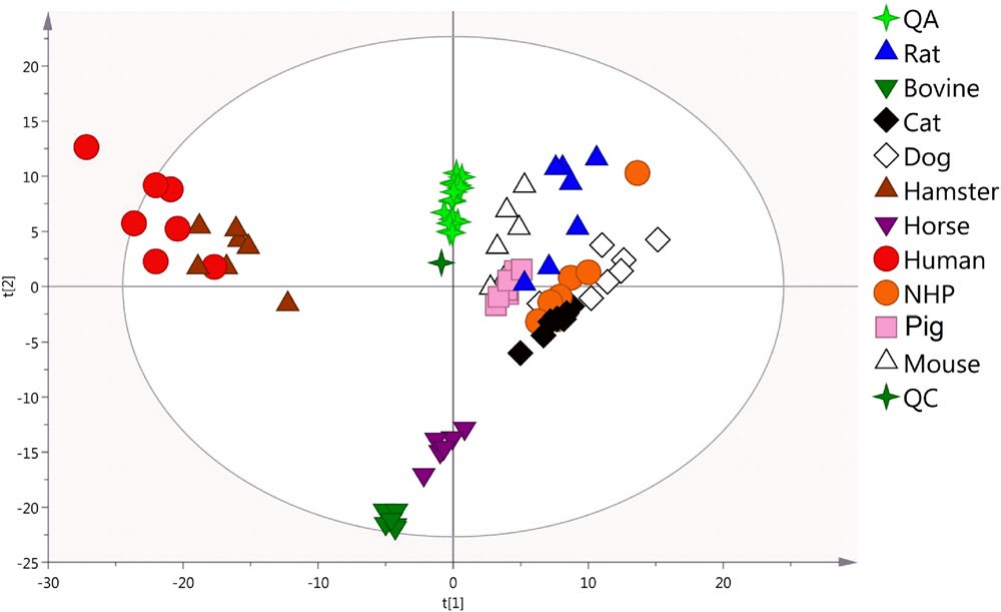
Comparison of non-targeted plasma lipid fingerprints of studied species. Principal component analysis (PCA) model based on 4,899 features extracted from the lipid fingerprints of human and non-human plasma samples (n = 10 × 6). R2X = 0.540; Q2 = 0.362. PC contributions: PC1 = 0.156, PC2 = 0.101.

A dataset of 4,899 variables common to all species was obtained from raw data. An unsupervised PCA model was applied to get an overview of variance between lipid profiles of species (Fig. 2). The analytical QA samples were well-clustered on the PCA model and plotted together with the biological QC sample attesting the quality of the analysis. In addition, the individual biological QC_species_ samples were plotted within each corresponding species plasma samples. This established PCA model explained 54% of the total variability and highlighted clustering between some animal species. Bovine and equine species (the herbivorous group), were characterized by a relatively low level of TG in line with biochemical measurements. This could likely explain their clustering and discrimination from the other species (Fig. 2). In sharp contrast, humans were plotted together with hamsters and were both well discriminated from the other species. Of note, no clear difference was observed between genders for each species. This underlined that sex differences were likely masked by those of species.

### Targeted lipidomics

**Figure 3:**
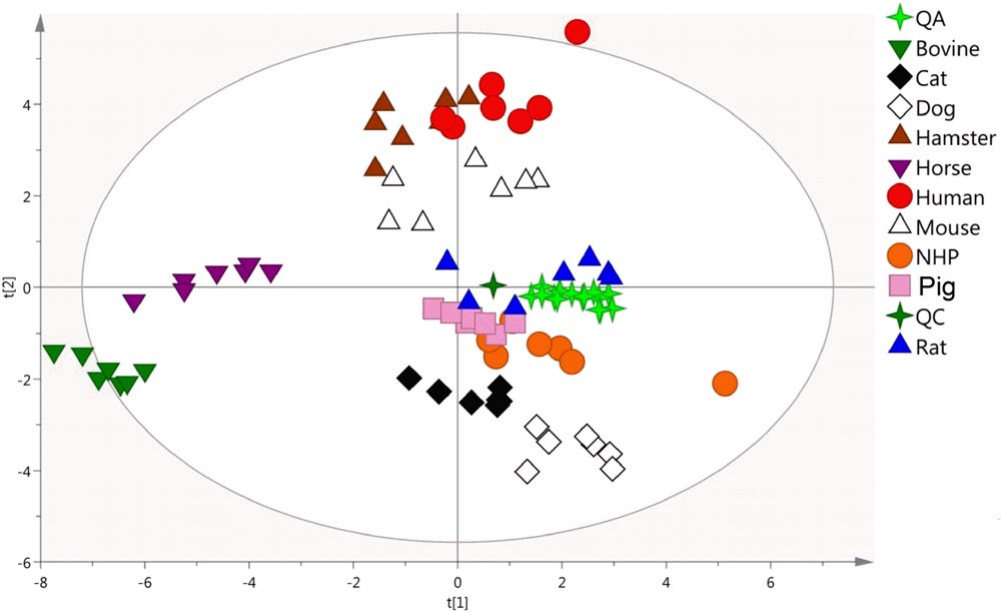
Comparison of targeted plasma lipid fingerprints of studied species. Principal component analysis (PCA) model based on 106 identified lipids from the lipid fingerprints of human and non-human plasma samples (n = 10 × 6). R2X = 0.744; Q2 = 0.577. PC contributions: PC1 = 0.242, PC2 = 0.153.

**Figure 4:**
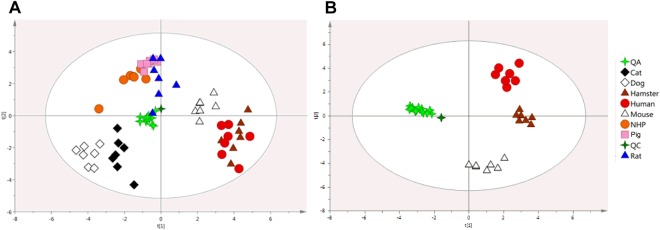
Comparison of targeted plasma lipid fingerprints of humans, mice and hamsters. (**A**) Principal component analysis (PCA) model based on 106 identified lipids from the lipid fingerprints of human and non-herbivorous animal species plasma samples (n = 8 × 6), R2X = 0.753; Q2 = 0.498. PC contributions: PC1 = 0.223, PC2 = 0.136. **(B)** PCA model based on 106 identified lipids from the lipid fingerprints of human, mouse and hamster plasma samples (n = 3 × 6), R2X = 0.776; Q2 = 0.698. PC contributions: PC1 = 0.302, PC2 = 0.169.

By querying a lipid library of 260 molecular species (Table 2), 106 were clearly and accurately identified from the 4,899 variables including CE, ceramides (Cer), SM, LPC, lysophosphatidylethanolamines (LPE), phosphatidylcholines (PC), phosphatidylethanolamines (PE), phosphatidylinositols (PI), diglycerides (DG), and TG (Supplemental Table S2). These lipids were subsequently used to build a new PCA model, exhibiting an acceptable clustering of QA and QC samples and explaining 75% of the total variability (Fig. 3). Similarly to the previous non-targeted PCA model, this one based on the 106 identified lipids showed a clustering of the different samples and individual QC_species_ for each species. Based on abundances of these 106 lipid markers, the intra-species gender difference was irrelevant in comparison to the inter-species lipid profile variance. In addition, bovine and equine plasma samples were again plotted together and clearly discriminated from the other study samples. Targeting a better understanding of the possible clustering between other species, an additional PCA model was established taking out bovine and equine species. This third PCA model (Fig. 4A) showed three separated groups of animals: 1) dog and cat; 2) pig, rat and NHP; 3) mouse, hamster and human. This targeted model also exhibited a clustering of mice along with hamsters and humans. Again, no difference was observed between genders for each species.

**Table 2:** Details of the in-house database used for lipid identification in positive ionization mode.

Classification	Number of lipids	Major adduct	RT range (min)
Fatty acyls			
Fatty esters	12	[M + H]^+^	0.51–4.12
Glycerolipids			
TG	77	[M + NH_4_]^+^, [M + Na]^+^	16.36–22.03
DG	6	[M + NH_4_]^+^, [M + Na]^+^	13.35–15.69
Glycerophospholipids			
LPE	5	[M + H]^+^	1.76–2.38
PE	16	[M + H]^+^	11.43–13.72
LPC	16	[M + H]^+^	1.45–3.85
PC	53	[M + H]^+^	8.93–15.83
PI	4	[M + NH_4_]^+^	6.91–12.56
Sphingolipids			
SM	30	[M + H]^+^	9.25–16.85
Cer	14	[M + H-H_2_O]^+^, [M+H]^+^	13.24–17.64
Sterol lipids			
CE	19	[M+NH_4_]^+^, [M + Na]^+^	18.54–20.17
Free sterols	8	M + H-H_2_O]^+^, [M + H]^+^	6.51–12.32
**TOTAL**	**260**		

RT, retention time; n/a, not applicable; TG, triglyceride; DG, diglyceride; LPE, lysophosphatidylethanolamine; PE, phosphatidylethanolamine; LPC, lysophosphatidylcholine; PC, phosphatidylcholine; PI, phosphatidylinositol; SM, sphingomyelin; Cer, ceramide; CE, cholesteryl ester.

### Comparison of mouse and hamster with human

**Figure 5:**
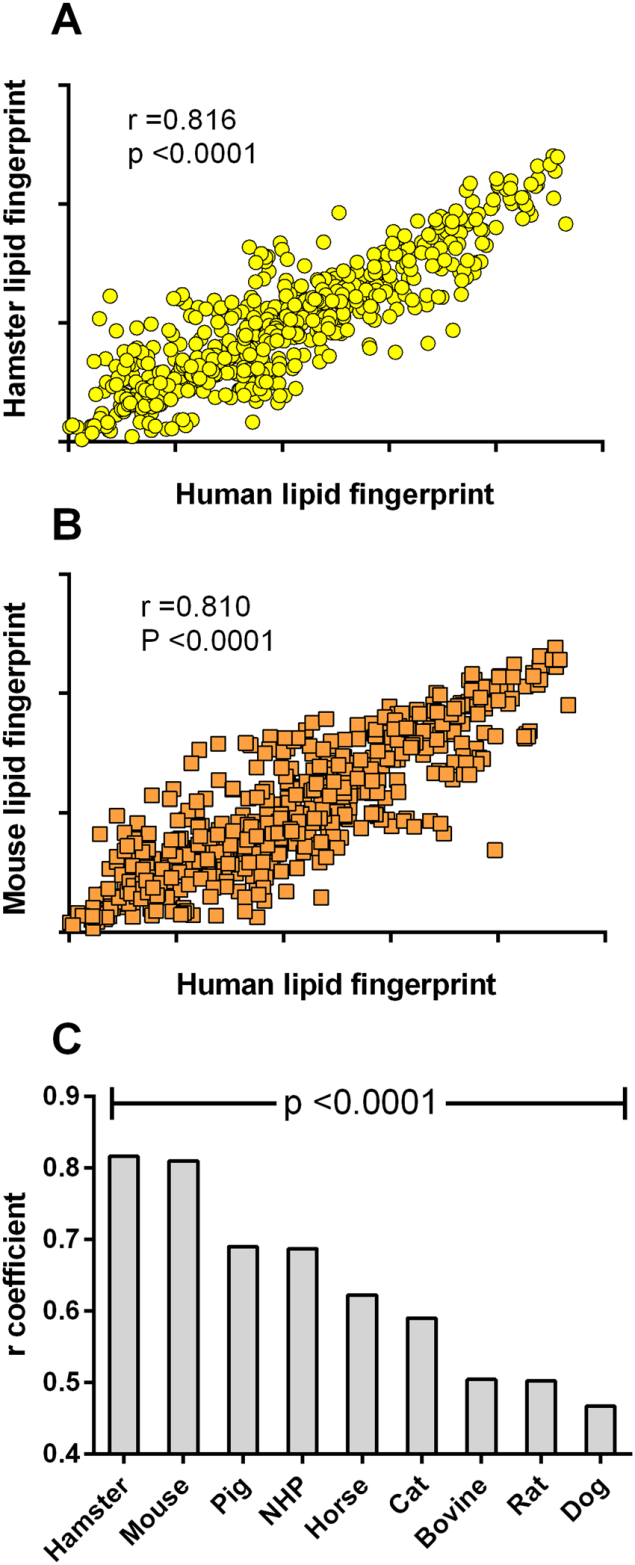
Correlation of lipid marker abundances between human and animals. (**A**) Pearson correlation obtained with the mean relative abundances (n = 6) of identified lipids (n = 106) **(A)** between hamster and human and **(B)** between mouse and human. **(C)** Pearson correlation coefficients (p < 0.0001) obtained with the relative abundances of identified lipids between human and each animal species (n = 106).

A fourth PCA model was built with only human, mouse and hamster (Fig. 4B) and this last one underlined again similarities between human and hamster. Univariate analyses were therefore carried out on global lipid class abundances between those species (Table 3). Further evaluations of the lipid profile similarity between humans and each of animal species were investigated. For each animal species, the relative abundances of the 106 lipids identified were compared to those obtained in human (Fig. 5). As anticipated, strong correlations were found between human and hamster (r = 0.816, p < 0.0001) or mouse (r = 0.810, p < 0.0001). For other species, r correlation coefficients were significant (p values range 0.001–0.05) with lower r coefficients (range 0.467–0.690).

**Table 3:** Mean fold changes (n = 6) of plasma lipid classes between human and hamster or mouse.

Lipid class	Number of species	Fold change (vs human)	
		Hamster	Mouse
Ceramides	6	0.78	0.50*
Sphingomyelins	5	0.40**	0.12**
Phosphatidylcholines	38	1.23	1.19
Phosphatidylethanolamines	7	0.95	1.02
Phosphatidylinositols	2	1.29	3.04**
Cholesteryl esters	5	0.68	3.03**
Diglycerides	3	0.32**	0.37**
Triglycerides	40	0.79	1.16

Values were compared using a Mann-Whitney comparison test (*p < 0.05, **p < 0.01).

## Discussion

Animal models are widely used to investigate and understand the physiopathology of diseases and to facilitate the development of treatments. Lipidomics helps to select relevant lipid biomarkers to target lipid disturbances and related ACVD. From a wide range of animal lipidomic phenotypes, we showed that hamster and, in a lower extent mouse, displayed similar lipid fingerprints compared with human beside traditional plasma lipids such as total cholesterol or triglycerides. This finding confirms these two species are convenient models to investigate some lipid disorders and related events.

We confirmed that human and pig lipoprotein profiles were similar and characterized by elevated LDL cholesterol compared to HDL cholesterol levels^13^. This is likely related to the physiological and anatomical similarities between both species^14^. With regard to rats and hamsters, similar lipoprotein profile analysis showed comparable distribution of cholesterol within HDL and LDL. Lower LDL cholesterol concentrations, compared with HDL cholesterol, were detected in these two species as in NHP, cat, mouse, dog, horse and cow in agreement with previous reports^15–18^. Elevated HDL cholesterol levels in mouse, dog, horse and cow could be closely related to a very low or not detected Cholesteryl Ester Transfer Protein (CETP) activity, which plays a critical role in lipid metabolism, especially by mediating cholesterol ester transfer from HDL to apolipoprotein B-containing lipoproteins (i.e. VLDL and LDL)^15,19^.

Lipid evaluation in ACVD risk is primarily focused on TC, TG, LDL and HDL cholesterol. However, detailed analyses of lipidome could improve risk prediction by targeting specific molecular species possibly involved in atherosclerosis or related diseases. In that respect, sphingomyelins were shown to be positively and independently related to the presence of coronary artery disease^20^ and lowering plasma ceramides was associated with decreased plasma cholesterol and inhibition of atherogenesis in apoE*3 Leiden and LDLR^−/−^ mice^21^. Epidemiological studies also revealed that plasma CE, DG, PE, LPE, LPC and PI species could be associated with cardiovascular diseases (CVD)^22,23^. More recently, PC metabolites were strongly associated with multiple CVD risk factors in teenagers^24^. In diabetic patients, a lipid signature including sphingolipids, phospholipids, sterols and glycerolipids, was associated with cardiovascular events and cardiovascular death^25^. Some similar lipid species were increased in apoE^−/−^ mouse plasma exhibiting atherosclerotic lesions^26^. These findings suggested that these lipid species could be powerful biomarkers for cardiovascular risk evaluation.

In that context, we performed a non-targeted analysis to give a global overview of similarities and differences between species. Our data unraveled a strong clustering between human and hamster which were well discriminated from the other species. From the main lipid classes found in plasma^27^, we identified 106 common molecular lipid species useful to better discriminate species. By targeting only these compounds, we showed that humans, mice and hamsters shared similar lipid profiles. This was reinforced by univariate analyses which revealed strong correlations between human and hamster or mouse unlike the other species such as dog and rat.

To date, only one study has investigated plasma lipid profile comparisons between healthy humans and animal species (rat, mouse and rabbit)^28^. From the 206 common identified lipids consisting of glycerophospholipids (LPC, LPE, PC, PE, PI), sphingolipids (SM, Cer, sulfatides), glycerolipids (DG) and sterol lipids (CE), abundances of 163, 166, and 151 metabolites were significantly different in mice, rats, and rabbits, respectively, when compared with humans^28^. In line with our data (Supplemental Table S2), the authors also showed lower sphingolipid abundances as well as elevated LPC and longer unsaturated PC levels in rodents in comparison to humans. Compared with human, our data showed that LPC were overall higher in animals (except for pig and horse), while PE and PI ranged similarly. We also noticed that PC carrying shorter or more saturated fatty acids (<C34, <3 double bonds) were significantly lower in animals. In contrast, PC carrying longer polyunsaturated acids (>C34, >3 double bonds) tended to be more abundant in animals than in humans. Noteworthy, our data clearly showed that hamster and human displayed similar PC profiles unlike other species (18 out of 33 PCs). Besides, it has been previously reported that PC (35:4) and PC (36:1) could be powerful markers involved in CVD^25^. PC (35:4) abundances were found similar between human and hamster, mouse, NHP, cat or bovine, while PC (36:1) abundances were close to those measured for human, mice and NHP but 2-fold higher in hamster. Except for dog, sphingolipid levels were significantly lower in animal species than in humans. However, human and animal species shared similar patterns for SM (d18:1/20:1) which was proposed as a precursor marker for CVD^29^, and no differences were shown between hamster and human regarding major ceramide species found in human plasma^30^. Triglycerides carrying shorter or less unsaturated fatty acids (<C54, <5 double bonds) were significantly lower in animal species than in humans except for hamsters. In contrast, TG carrying higher and polyunsaturated fatty acids (>C54, >5 double bonds) were more abundant in animal species than in humans. However, our results highlighted that hamsters and mice displayed a TG profile closer to that of humans than others species (36 out of 44 TGs). No significant difference was found between hamsters or mice and humans regarding to TG (54:2) which was identified as most consistently linked to CVD^23^. Human and animal species significantly displayed different levels of CEs likely due to the inter-species modulations of CETP. Again, no significant differences were observed between hamsters and humans regarding to CE (16:1) which has been recently associated with CVD^25^.

A limitation of the study is the small number of samples per species (6 individuals). However, we aimed to extend the analysis to a maximum of animal species with an appropriate group size to obtain meaningful multivariate analyses. Another hurdle could be the selection of 3 males and 3 females per animal species as lipid phenotypes are well-known to be impacted by gender. We chose this option to underline inter-species differences in lipid fingerprints independently of gender. In that respect, our data showed that sex differences were minimal compared to those of species. Nevertheless, this suggests that further analyses are warranted in this field.

In summary, this is the first study that explored molecular lipid species in plasma from humans and nine animal species besides usual plasma lipids. We showed that hamster and human lipid profiles were comparable beside cholesterol and TG. We also identified 89 and 102 common molecular species between human and mouse or hamster, respectively. In contrast to mice, hamster and human also shared 13 common lipids belonging to Cer, PI and CE classes, which can be used to identify the metabolic variation associated with early atherosclerosis and to select biomarkers for ACVD^11^. Of note, it has also been shown that hamsters are able to develop a diet-induced atherogenesis with a close similarity to the human lipoprotein profile and foam cell formation^28–35^, while transgenic mice are required to investigate such physiological aspects (mainly apoE or LRL receptor knockout mice)^12^. Finally, hamsters also share with humans an exclusively hepatic and intestinal production of apolipoprotein B100 and B48, respectively^35^, as well as a CETP activity. Reflecting the human setting, the hamster appeared to be the most relevant model to monitor a time-dependent development of atherosclerosis and to identify plasma lipids involved in underlying mechanisms.

## Methods

### Plasma samples and study design

Plasma EDTA samples were obtained from 10 species (Table 1) namely human, non-human primate (NHP), dog, cat, pig, horse, bovine, hamster, mouse and rat. Samples were collected from healthy and overnight fasted males (n = 3) and females (n = 3) at ONIRIS (Veterinary school of Nantes, France). Plasma collections were conducted in agreement with the animal welfare guidelines and approval of ONIRIS Ethics Committee. Human plasma samples were collected at the Nantes hospital. All of the participants provided written informed consent, and the study was approved by the local human Ethics committee (Nantes Hospital, France). All of the methods were performed in accordance with the relevant guidelines and regulations. All samples were stored at −80 °C prior to analysis that was performed in the month following collection.

### Lipoprotein profiling and biochemical analysis

Plasma lipoprotein fractions, including very low-density lipoprotein (VLDL), low-density lipoprotein (LDL) and high-density lipoprotein (HDL), were separated by Fast Protein Liquid Chromatography (FPLC, Amersham Pharmacia Biotech Inc., Orsay, France) as described previously^36^. TC, TG, cholesteryl esters (CE) and phospholipids (PL) were assayed in plasma and FPLC fractions using enzymatic test kits from Boehringer Mannheim GmbH (Mannheim, Germany).

### Non-targeted lipidomic analysis

All solvents were purchased from Biosolve (Valkenswaard, Netherlands). The non-targeted analysis was performed on the full set of plasma samples (n = 6 individuals × 10 species). One quality control sample (QC_species_) per species was prepared by pooling 5 µL from each individual sample of the same species. Then, a quality control sample (QC) was formed by pooling 5 µL aliquots from each QC_species_ sample, and subsequently split in two 25 µL aliquots. Lipids were extracted from plasma and QC samples (25 µL) as reported previously^37^. In brief, 225 µL of ice-cold methanol were initially added to defrost plasma samples. Following 10 seconds vortex, 750 µL of ice-cold methyl-*ter*-butyl ether (MTBE) containing exogenous internal standards [10 µmol/L; Cer (d18:1/17:0), SM (d18:1/17:0), CE (19:0), *d*_31_-LPC (16:0), *d*_62_-PC (32:0), *d*_62_-PE (32:0), PI (31:1), and *d*_5_-TG (50:0); Avanti Polar Lipids, Inc., Alabaster, AL, USA] were added and the mixture was vortexed for 10 seconds. Finally, 188 µL of water were added and vortexed for 10 seconds. The final mixture was centrifuged (10,000 *g*; 10 minutes, 5 °C) and 600 µL of supernatant were transferred to LC-MS vial to be evaporated to dryness under a nitrogen stream (room temperature). Dried samples were reconstituted with 110 µL mixture of acetonitrile/isopropanol/water (65/30/5, v/v/v). A quality assurance sample (QA) was prepared by pooling 10 µL from each vial. This QA sample was injected throughout the analytical batch for correction and normalization purposes^38^. Ultra-performance liquid chromatography-high-resolution mass spectrometry (UPLC-HRMS) analyses were performed on a Synapt^TM^ G2 HRMS Q-TOF mass spectrometer equipped with an electrospray ionization (ESI) interface operating in the positive ionization mode, and an Acquity H-Class^®^ UPLC^TM^ device (Waters Corporation, Milford, MA, USA). Samples were randomized and injected (5 µL) altogether with QA extracts onto a reversed-phased LC column. Lipids were eluted as detailed in the Supplemental Table S1. The total number of samples was: 6 individuals by 10 species, 1 QC_species_ by 10 species, 1 QC and 1 QA repeatedly injected throughout the batch. The full-HRMS mode was applied for lipid detection (mass-to-charge ratio (*m/z*) range 200–1,200) at a mass resolution of 25,000 full-widths at half maximum (continuum mode). The ionization settings were as follows: capillary voltage, +2 kV; cone voltage, 30 V; desolvation gas (N_2_) flow rate, 900 L/h; desolvation gas/source temperatures, 550/120 °C. Leucine enkephalin solution at 2 µg/mL (50% acetonitrile) was infused at a constant flow rate of 10 µL/min in the lockspray channel, allowing for correction of the measured *m/z* throughout the batch (theoretical *m/z* 556.2771). Data acquisition was achieved using MassLynx^TM^ software version 4.1 (Waters Corporation).

### Data processing

Raw files initially processed with MassLynx software were converted to mzXmL format with MSConvert version 3.0.3347 (Vanderbilt University, Nashville, TN, USA). The converted raw data were processed by XCMS software version 1.38.0 running under R version 3.2.0. The Matchedfilter algorithm was used with following parameters: step = 0.03, fwhm = 10, snthresh = 10 and mzdiff = 0.01. Furthermore, “Obiwarp” method was selected for peaks alignment and “group density” function was used for grouping peaks (bw = 9, mzwid = 0.008, minsamp = 5 and max = 30). The function “Fill Peaks” was discarded. Consequently, all non- integrated peaks by XCMS software were affected with Non Attributed (NA) annotation in the generated dataset table. The features presenting “NA” annotations or zeros were processed in a manner to replace the “NA” annotations or the zeros of each feature with randomly generated values ranging between −30% and +30% of the lowest detected signal of each corresponding feature, avoiding hence artificial zero abundance values. A filtration step was automatically performed aiming at eliminating the different isotopomers (isotopic pattern) (M + 1, M + 2 and M + 3) and possible adducts (sodium, potassium, ammonium acetate, sodium ammonium acetate and acetonitrile) of a same compound. Such a filtration was performed using a home-made informatics tool taking into account the two following criteria: a maximum mass difference of 3 mDa and a maximum retention time variation of ±2 seconds with the compound ion adduct. Finally, features exhibiting a CV > 30% in the QA batches were discarded for further data analysis step. Relative abundance of variables (putative lipid markers) was obtained from peak areas normalized to exogenous internal standards. Lipid markers were then extracted from the detected variables using an in-house database containing 260 reference lipid standards, their exact mass measured, their elemental compositions with a mass error of ±5 ppm, their retention times (±30 seconds), and their fragmentation patterns in tandem mass spectrometry (Table 2).

### Statistical analysis

Targeting a comprehensive comparison of our sample profiles originating from the different human and animal species, unsupervised “Principal Component Analysis” (PCA) models were used with dedicated software (SIMCA-P+, version 13.0, Umetrics, Umea, Sweden). A logarithmic transformation and Pareto scaling were applied to generate unsupervised PCA giving a general overview of the main discriminations and assessing the analytical robustness through the QA samples clustering. Models validity was appraised using permutation tests and CV-ANOVA. Univariate statistical analyses were performed with GraphPad Prism software (version 6.0, GraphPad Software Inc., La Jolla, CA, USA). Correlations were carried out using the parametric Pearson test and groups were compared using the Mann-Whitney test. Results were considered significant at p < 0.05.

## Electronic supplementary material

Supplemental materials

## Data Availability

The datasets analyzed during the current study are available from the corresponding author on reasonable request.
