# Supplementary material for: Plasma lipidomic analysis reveals strong similarities between lipid fingerprints in human, hamster and mouse compared to other animal species
Source: Sci Rep. 2018 Oct 26;8:15893. doi: 10.1038/s41598-018-34329-3 (PMC6203725; doi:10.1038/s41598-018-34329-3)
Supplement: Supplementary file 1 — Supplemental materials [file 41598_2018_34329_MOESM1_ESM.pdf]

Plasma lipidomic analysis reveals strong similarities between lipid fingerprints in human, hamster and mouse compared to other animal species

Zied Kaabia, Michelle Moughaizel, Julie Poirier, Audrey Aguesse, Stéphanie Billon-Crossouard, Fanta Fall, Manon Durand, Elie Dagher, Michel Krempf, and Mikaël Croyal

SUPPORTING INFORMATION

Supplemental Table S1 - Details of the elution methods.

Methods	Time (min)	Mobile phase A (%)	Mobile phase B (%)
Acquity® CSH C_{18} column<br>2.1 × 100 mm, 1.7 µm<br>Flow: 300 µL/min<br>Oven T°: 30 °C<br>Tray T°: 4°C	0.0	60	40
	1.0	60	40
	2.0	50	50
	12.0	30	70
	18.0	1	99
	21.0	1	99
	22.0	60	40
	25.0	60	40

Mobile phase A: 60/40 (acetonitrile/water, v/v) + 0.1% acetic acid + 10 mM ammonium acetate – Mobile phase B: 88/10/2 (isopropanol/acetonitrile/water, v/v/v) + 0.1% acetic acid + 10 mM ammonium acetate.

Supplemental Table S2 – Relative abundances of plasma lipids identified in this study. Values are the mean raw intensities normalized to those obtained in human. Bold indicates significant differences ($p < 0.05$) evaluated with a Mann-Whitney comparison test.

**Lipids**	**Hamster**	**Mouse**	**Pork**	**NHP**	**Horse**	**Cat**	**Bovine**	**Rat**	**Dog**
Ceramide									
Cer (d18:1/16:0)	0.89	**0.45**	**2.67**	0.83	**0.21**	1.03	**0.37**	0.93	1.58
Cer (d18:1/18:0)	**0.29**	**0.19**	**3.01**	**0.49**	**0.22**	1.29	**0.18**	**0.39**	**10.6**
Cer (d18:1/22:0)	**0.63**	**0.57**	**0.40**	**0.48**	**0.28**	**0.42**	**0.03**	**0.49**	1.20
Cer (d18:1/22:1)	0.57	**0.49**	**0.34**	0.77	**0.15**	**0.29**	**0.14**	**0.33**	0.64
Cer (d18:1/24:1)	0.92	0.69	0.60	0.53	**0.18**	0.51	**0.03**	0.82	1.48
Cer (d18:1/25:0)	**0.54**	**0.12**	**0.08**	0.61	**0.21**	**0.42**	**0.07**	1.04	**3.83**
Sphingomyelin									
SM (d18:1/16:0)	**0.61**	**0.12**	**0.43**	**0.50**	**0.38**	**0.98**	**0.49**	**0.18**	**5.19**
SM (d18:1/20:0)	**0.11**	**0.04**	**0.28**	**0.08**	**0.39**	**0.83**	**0.04**	**0.08**	9.74
SM (d18:1/20:1)	0.98	0.87	0.94	0.91	**1.74**	0.97	**1.73**	0.94	0.95
SM (d18:1/22:0)	**0.15**	**0.15**	**0.07**	**0.09**	**0.23**	**0.60**	**0.08**	**0.25**	**2.14**
SM (d18:1/24:1)	**0.21**	**0.12**	**0.08**	**0.43**	**0.07**	**0.34**	**0.04**	**0.29**	**4.12**
Lyso-phosphatidylcholine									
LPC (16:0)	**1.90**	**1.59**	**0.26**	0.60	**0.03**	**0.41**	**0.05**	1.25	**0.27**
LPC (20:1)	1.26	1.01	1.09	1.06	0.99	0.95	1.11	**1.72**	1.00
LPC (20:4)	0.97	0.85	0.91	1.15	1.25	**1.48**	1.25	0.93	1.22
LPC (20:5)	**1.56**	**1.81**	**0.23**	1.31	**0.12**	0.94	**0.07**	**1.38**	0.57
LPC (22:4)	**3.90**	**3.64**	1.61	**2.68**	0.69	**2.36**	**2.12**	**4.94**	**3.25**
Lyso-phosphatidylethanolamine									
LPE (20:4)	6.61	13.95	0.59	0.54	0.57	1.04	0.61	0.88	0.05
Phosphatidylcholine									
PC (32:0)	0.70	0.89	**0.42**	1.27	**0.47**	0.64	**0.38**	1.03	0.67
PC (32:0e)	0.77	**0.34**	0.79	0.94	**0.72**	1.11	**0.23**	**0.53**	1.43
PC (32:0p)	**0.25**	**0.13**	**0.22**	**0.69**	**0.64**	**0.47**	**0.61**	**0.20**	**0.69**
PC (32:1)	0.68	1.19	**0.29**	**0.25**	**0.55**	**0.34**	**0.29**	**0.30**	**0.26**
PC (34:1)	1.28	1.08	**0.52**	**0.39**	**0.53**	**0.58**	**0.37**	**0.33**	**0.55**
PC (34:1e)	0.88	**0.33**	1.18	0.55	**1.81**	**2.81**	0.60	**0.45**	**2.31**

**Lipids**	**Hamster**	**Mouse**	**Pork**	**NHP**	**Horse**	**Cat**	**Bovine**	**Rat**	**Dog**
PC (34:3) iso 1	**0.31**	**0.08**	**0.08**	**0.08**	**0.07**	**0.08**	**0.08**	**0.08**	**0.80**
PC (34:3) iso 2	**0.28**	**0.20**	**0.17**	**0.18**	**0.17**	**0.18**	**0.20**	**0.19**	**0.82**
PC (34:3) iso 3	**1.65**	**4.50**	0.99	2.56	0.80	**6.45**	**0.64**	**2.11**	**3.46**
PC (34:4)	**0.27**	**0.45**	**0.52**	1.08	**0.19**	**0.48**	**0.39**	**3.16**	**3.42**
PC (35:4)	0.88	0.76	**0.55**	2.54	**0.56**	1.45	1.19	**3.63**	**4.76**
PC (36:0)	0.90	**1.42**	**0.77**	1.43	0.91	1.69	0.85	**4.12**	0.58
PC (36:1)	**1.93**	0.92	**2.30**	0.64	**2.41**	**4.58**	**3.09**	**0.39**	**3.45**
PC (36:2e) iso 1	1.40	**0.19**	1.50	0.63	**3.08**	1.75	0.65	**0.19**	**2.33**
PC (36:2e) iso 2	**0.40**	**0.27**	0.49	**0.30**	**0.26**	0.49	**0.27**	**0.30**	0.83
PC (36:3)	0.82	**1.53**	**0.22**	**2.21**	**0.27**	0.73	1.00	**0.25**	0.58
PC (36:3e)	0.73	**0.63**	1.14	0.99	**4.79**	**2.53**	**0.44**	**0.24**	**3.93**
PC (36:3p)	0.56	0.54	0.57	1.18	1.91	0.86	0.55	0.56	1.09
PC (36:4)	0.74	**3.65**	0.76	**12.53**	**6.88**	**5.14**	**0.20**	2.20	**1.87**
PC (37:2)	**17.19**	**13.36**	0.87	**8.95**	**15.26**	**18.4**	**3.95**	2.61	**10.7**
PC (38:4)	**1.40**	**1.28**	**4.98**	**2.42**	**0.46**	**7.37**	1.06	**8.81**	**7.68**
PC (38:4p)	1.02	0.98	1.07	2.63	0.95	1.02	1.98	1.06	1.14
PC (38:5)	**2.47**	**2.66**	**4.46**	**2.60**	**0.37**	**6.80**	**1.27**	**3.63**	**4.36**
PC (40:4)	1.24	0.97	**19.78**	**9.83**	0.70	**18.1**	**5.93**	**5.19**	**24.8**
PC (40:4e)	**0.20**	**0.37**	**0.53**	0.69	**0.17**	1.22	**0.13**	**0.36**	1.69
PC (40:5) iso 1	**0.64**	1.24	**16.82**	**5.20**	1.36	**8.35**	**19.73**	**4.64**	**27.8**
PC (40:5) iso 2	**13.76**	**2.44**	**5.99**	**8.60**	**0.66**	**7.26**	0.75	**17.0**	1.09
PC (40:5e) iso 1	0.14	0.34	1.31	**3.78**	0.21	0.10	1.01	0.32	**15.9**
PC (40:5e) iso 2	1.21	1.06	1.11	**1.80**	1.01	**1.77**	1.08	1.14	1.10
PC (40:6)	0.88	0.93	0.88	0.97	1.07	1.04	**1.80**	0.92	3.48
PC (40:7) iso 1	**0.79**	1.00	0.84	0.97	0.87	**2.02**	**0.85**	0.93	1.18
PC (40:7) iso 2	0.97	1.06	1.08	1.05	1.13	1.04	1.11	0.95	**6.84**
PC (42:5e)	**0.09**	**0.11**	**0.17**	**0.34**	**0.10**	**0.27**	**0.07**	**0.11**	0.99
Phosphatidylethanolamine									
PE (36:1) iso 1	1.06	1.05	1.07	1.43	1.05	1.06	1.08	1.00	0.88
PE (36:1) iso 2	1.08	1.04	0.99	1.03	1.12	1.06	1.44	1.01	0.88
PE (38:4)	0.88	0.76	**0.55**	2.54	**0.56**	1.45	1.19	**3.63**	**5.44**
PE (38:6)	0.90	1.01	1.02	1.00	**1.47**	1.04	0.91	0.91	0.77
PE (40:6)	1.05	1.04	**1.21**	**13.13**	1.09	1.05	1.03	1.26	**1.85**
PE (40:7)	1.05	1.05	0.91	1.06	0.88	1.06	1.01	1.08	**1.18**

**Lipids**	**Hamster**	**Mouse**	**Pork**	**NHP**	**Horse**	**Cat**	**Bovine**	**Rat**	**Dog**
Phosphatidylinositol									
PI (34:1)	1.61	**4.95**	**1.27**	2.33	1.11	**2.10**	1.12	**1.53**	0.47
PI (36:2)	0.99	1.19	0.98	**2.42**	1.02	**1.64**	1.18	1.16	0.94
Diglyceride									
DG (34:1)	**0.46**	**0.31**	0.97	**0.36**	**0.16**	**0.12**	**0.07**	0.67	**0.10**
DG (36:1)	**0.22**	**0.47**	**2.14**	1.69	**0.20**	**0.62**	**0.06**	3.32	**0.59**
DG (36:4)	**0.31**	0.79	**2.89**	**10.13**	0.68	**2.15**	**0.30**	**17.2**	**2.04**
Triglyceride									
TG (40:0)	0.31	0.17	0.28	0.19	0.15	0.15	0.19	9.94	0.16
TG (44:0)	0.75	0.42	1.06	0.32	0.34	0.35	0.35	0.71	0.37
TG (44:0)	0.91	1.01	0.91	1.15	**0.77**	0.83	1.16	0.85	1.01
TG (44:1)	0.53	**0.21**	0.86	**0.21**	**0.21**	**0.13**	**0.20**	1.42	**0.29**
TG (46:0)	0.94	**0.38**	0.89	**0.47**	0.75	**0.32**	**3.45**	0.92	**0.44**
TG (46:1)	0.72	**0.42**	0.86	**0.23**	**0.36**	**0.11**	**0.36**	1.16	**0.30**
TG (47:0)	**3.00**	**0.54**	**0.70**	0.87	0.72	**0.54**	**6.15**	1.11	0.72
TG (48:0)	0.85	0.91	1.35	0.67	0.69	**0.12**	0.60	0.56	0.43
TG (48:1)	0.75	1.26	0.92	**0.55**	**0.36**	**0.10**	**0.06**	**0.47**	**0.28**
TG (48:2) iso 1	0.55	1.30	0.95	**0.53**	**0.31**	**0.08**	**0.10**	**0.45**	**0.16**
TG (48:2) iso 2	0.55	1.30	0.95	**0.53**	**0.31**	**0.08**	**0.10**	**0.45**	**0.16**
TG (48:3)	1.10	0.90	**2.55**	1.04	1.27	1.06	0.90	5.40	0.97
TG (49:0)	1.06	1.02	**1.41**	0.95	1.05	0.95	1.07	1.11	1.12
TG (49:2)	1.02	0.90	1.11	1.58	**0.51**	**0.25**	0.71	0.68	**0.41**
TG (49:3)	0.94	1.24	0.77	0.76	0.82	0.82	0.91	0.85	0.79
TG (50:1)	0.87	1.46	1.08	1.05	**0.45**	**0.20**	**0.06**	0.69	**0.31**
TG (50:2)	0.85	1.36	1.11	1.05	**0.36**	**0.13**	**0.05**	0.69	**0.18**
TG (50:3)	0.60	1.82	**1.45**	0.90	0.60	**0.33**	**0.07**	0.68	**0.27**
TG (51:3)	0.67	0.92	**1.96**	**1.81**	**0.46**	**0.53**	**0.42**	0.90	**0.55**
TG (51:4)	0.80	0.95	0.99	4.93	**0.35**	0.83	**0.35**	1.08	0.72
TG (52:0)	**6.06**	1.37	1.23	0.89	1.00	0.94	0.95	0.90	0.98
TG (52:1)	1.14	1.16	0.96	**0.33**	**0.12**	**0.31**	**0.02**	0.68	**0.15**
TG (52:3)	0.77	0.97	1.05	1.04	**0.21**	0.76	**0.03**	1.16	**0.51**
TG (52:5)	**0.33**	**2.73**	**8.99**	**7.58**	2.39	2.06	**0.13**	3.00	**0.97**
TG (53:1) iso 1	1.30	0.65	1.11	**0.36**	**0.30**	**0.26**	**0.18**	**0.49**	**0.31**
TG (53:1) iso 2	**2.67**	0.90	1.09	0.99	0.96	1.05	1.10	0.97	1.04

**Lipids**	**Hamster**	**Mouse**	**Pork**	**NHP**	**Horse**	**Cat**	**Bovine**	**Rat**	**Dog**
TG (54:1)	0.88	1.14	**2.37**	**0.47**	**0.23**	**0.36**	**0.63**	0.64	**0.50**
TG (54:2)	1.00	1.30	**3.03**	**0.41**	**0.20**	**0.36**	**0.15**	0.95	0.37
TG (54:3)	1.68	0.69	0.77	0.77	0.15	0.36	0.05	0.71	0.49
TG (54:4)	0.43	1.14	**3.45**	2.16	0.43	1.04	**0.09**	**2.90**	1.22
TG (54:5)	0.23	1.48	**5.21**	**6.19**	1.23	**2.25**	**0.11**	**5.63**	1.29
TG (54:7)	0.90	**0.52**	**0.32**	**0.39**	**0.40**	**0.49**	**0.46**	**0.38**	**0.39**
TG (55:1)	**25.90**	**2.83**	0.88	0.83	0.92	0.62	**17.05**	**3.09**	2.05
TG (56:0)	**3.37**	1.59	0.77	0.59	0.45	0.41	0.43	2.07	0.56
TG (56:1)	**3.73**	1.69	0.76	0.52	0.42	**0.19**	**3.43**	2.29	0.31
TG (56:2)	2.02	**3.83**	1.16	0.54	**0.34**	**0.24**	0.53	2.59	0.15
TG (56:4)	0.85	0.88	0.78	0.69	0.67	1.92	0.73	0.68	0.81
TG (56:6)	1.30	0.75	**8.29**	**6.57**	0.69	**4.85**	0.48	**21.5**	**10.3**
TG (56:7)	**2.25**	6.06	**10.96**	**8.31**	0.43	**6.31**	**0.26**	**31.5**	**13.3**
TG (58:5)	1.13	1.10	1.42	2.10	1.44	1.04	1.14	1.92	1.22
TG (60:1)	1.51	1.15	0.99	0.89	1.01	1.15	1.00	**4.49**	0.97
TG (60:2)	1.07	1.92	0.96	1.15	1.16	0.96	1.12	**5.15**	1.03
TG (60:3)	1.03	1.26	1.01	1.30	0.99	1.02	0.91	1.43	0.90
TG (60:11)	0.85	0.97	0.93	1.04	1.04	1.00	0.93	12.1	1.06
Cholesteryl ester									
CE (16:1)	1.28	**2.13**	**0.47**	**0.32**	**0.53**	**0.51**	0.92	**0.48**	**0.50**
CE (18:2)	**0.58**	0.79	0.75	**1.97**	**1.99**	**1.82**	1.16	**0.41**	**2.52**
CE (20:3)	1.20	**2.65**	**0.50**	**2.00**	**0.36**	**2.46**	1.71	1.27	**2.51**
CE (22:5)	**5.11**	**5.22**	**3.57**	**4.35**	0.61	**4.43**	**5.73**	**8.48**	**7.52**
CE (22:6)	**0.42**	**8.83**	**0.54**	**3.17**	**0.04**	**2.09**	**0.29**	**2.90**	**3.64**
